# Collaborative approaches for Target ID in neglected diseases: identifying new antimalarial targets

**DOI:** 10.1186/1475-2875-13-S1-P54

**Published:** 2014-09-22

**Authors:** Maria Jose Lafuente, Sonja Guidelli-Disse, Carolyn Selenski, Joel Lelievre, Timothy Willson, Francisco Javier Gamo, Gerard Drewes, Omar Vandal, Luis Ballell-Pages

**Affiliations:** 1 DDW Malaria DPU GSK, Tres Cantos, Madrid, Spain; 2 Cellzome GSK, Heidelberg, Germany; 3 R&D Platform Technology & Science, North Carolina, USA; 4 The Bill and Melinda Gates Foundation, Seattle, USA

## 

Malaria is a global health problem that causes significant mortality and morbidity, with more than 1 million deaths per year caused by *P. falciparum*. Most antimalarial drugs face decreased efficacy due to the spread of resistance. Most of the current compounds used for malaria treatment share related mechanisms of action so, there is an urgent need to identify new tractable targets to enable next generation of antimalarial drugs.

The malaria drug discovery community has embraced phenotypic screening approaches as the strategy of choice for the generation of novel chemical starting points for Lead Optimization programs. Determining the mode of action for novel whole cell hits remains a challenge, but new technologies, including genetics, chemoinformatics and proteomics are proving successful.

A chemical proteomics platform aimed at identifying antimalarial targets has been established. This platform is supported by The Bill & Melinda Gates Foundation and encompasses chemical probe synthesis, biological material preparation, pull down experiments and LC-MS protein-lead family characterization for chemical families of interest. Candidate scaffolds to be investigated through this platform are selected based on their biological profile by a join Steering Committee constituted by both GSK and BMGF members.

